# Severe Murine Typhus Presenting with Acalculous Cholecystitis: A Case Report and Literature Review

**DOI:** 10.1155/2017/3769074

**Published:** 2017-04-04

**Authors:** Nikolaos Spernovasilis, Constantinos Tsioutis, Maria Zafeiri, Georgios Hamilos, Achilleas Gikas

**Affiliations:** ^1^Internal Medicine Department, Infectious Diseases Unit, University Hospital of Heraklion, Heraklion, Crete, Greece; ^2^School of Medicine, European University Cyprus, Nicosia, Cyprus; ^3^Department of Medicine, University of Crete, Heraklion, Crete, Greece

## Abstract

A 54-year-old otherwise healthy male, who was being evaluated for prolonged fever, developed clinical and ultrasonographic signs compatible with acute acalculous cholecystitis. Diagnosis of murine typhus was confirmed by serology and the patient was treated with doxycycline. He improved rapidly and all clinical and laboratory abnormalities returned to normal. The present case dictates that knowledge of the local epidemiology and keeping a high index of clinical suspicion can help recognize uncommon manifestations of murine typhus, in order to treat appropriately and avoid unnecessary investigations and interventions.

## 1. Introduction

*R. typhi*, previously known as* R. mooseri*, belongs to the typhus group of rickettsiae (together with* R. prowazekii* which causes epidemic typhus) [1–3]. The main vector is the rat flea* Xenopsylla cheopis*, although other arthropods, such as the cat flea* Ctenocephalides felis*, have been implicated in the life cycle of* R. typhi* [1–3]. Humans are considered accidental hosts, primarily contaminated by inoculation of the rickettsiae through a fleabite site on their skin [2, 3]. Although history of a fleabite is not usually recalled, humans are typically infected in areas where close contact with animals and their fleas are most likely to occur [4].

Murine typhus is an endemic infection in tropical and subtropical seaboard regions throughout the world, including the Mediterranean [2]. In Greece, the disease was first described in 1932 [5]. Since then, endemic cases have been reported on the Greek islands of Euboea and Crete [5]. Here, we present a case of severe murine typhus presenting as acalculous cholecystitis in an otherwise healthy man.

## 2. Case Presentation

A 54-year-old Caucasian male was referred from Kasos island to the infectious diseases unit of our hospital with a 10-day history of fever, throbbing frontal headache, chills, and malaise. The patient was already receiving amoxicillin-clavulanate for suspected sinusitis, without apparent improvement. He lived in a rural area, worked as a cafeteria owner, and had frequent contact with animals (pigs and chicken) and bees. His past medical history was significant only for heterozygous *β*-thalassemia.

Upon presentation, the patient appeared ill, with a temperature of 38°C, resting blood pressure 110/55 mmHg, heart rate 85 beats per minute, respiratory rate 18 breaths per minute, and oxygen saturation 93% on room air. Auscultation revealed mildly prolonged expiration with no additional sounds, while mild right upper quadrant (RUQ) tenderness was noted. A rash was difficult to detect due to his dark complexion. The remainder of the examination was normal. There was evidence of lymphopenia (1 × 10^9^/L, reference range [RR] 1.5–3.6 × 10^9^/L), mild anemia (hemoglobin, 12.3 g/dL [RR 14–18 g/dL]; MCV, 62.9 fl [RR 80–99 fl]), and thrombocytopenia (131 × 10^9^/L [RR 150–450 × 10^9^/L]). In the peripheral blood smear, Howell-Jolly bodies were observed. Additional workup revealed mild hyponatremia (130 mEq/L [RR 135–145 mEq/L]), mild elevation of alanine aminotransferase (ALT, 51 U/L [RR 8–40 U/L]), aspartate aminotransferase (AST, 60 U/L [RR 8–40 U/L]), lactate dehydrogenase (LDH, 379 U/L [RR 80–230 U/L]), and a high C-reactive protein (13.2 mg/dL, normal < 0.8 mg/dL). Urine testing showed mild proteinuria, red blood cells (6/HPF), white blood cells (9/HPF), and a bland urine sediment. Arterial blood gas analysis on ambient air showed mild hypoxemia (pH 7.44, PaO_2_ 70 mmHg, and PaCO_2_ 33 mmHg), but no abnormalities were apparent on chest X-ray. The patient was placed off the antibiotics that he was taking, blood and urine cultures were drawn, and serology was obtained for viral infections (HIV, hepatitis B and C viruses, CMV, and EBV) and zoonoses (*Brucella* spp.,* Rickettsia* spp.,* Coxiella burnetii*,* Leishmania* spp., and leptospirosis) because of his epidemiological history.

**Figure 1:**
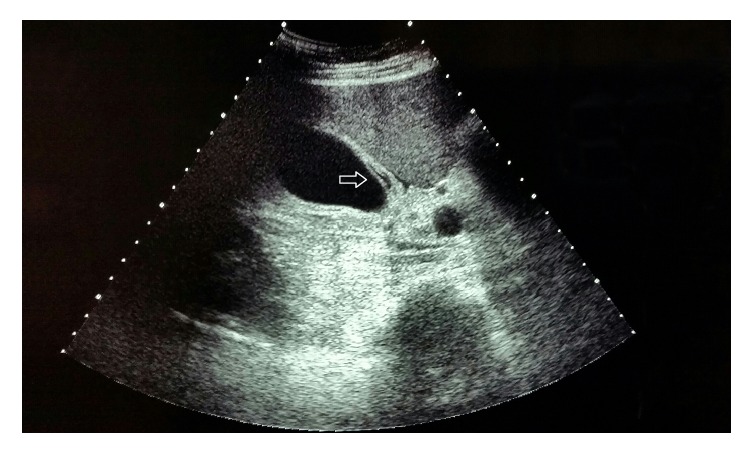
Ultrasound of the gallbladder, revealing thickened gallbladder wall with a layered appearance (arrow).

On the second day of hospitalization, while still febrile, the patient developed acute RUQ pain, with elevation of liver enzymes (ALT 61 U/L; AST 89 U/L; LDH 535 U/L). Abdominal ultrasound revealed thickened gallbladder wall with a layered appearance and mild pericholecystic fluid (Figure 1), while a positive ultrasonographic Murphy's sign was induced by the ultrasound probe. Subsequently, the patient was started on ceftriaxone plus metronidazole. On day 3, indirect immunofluorescence (IFA) obtained on admission revealed a high titer for* R. typhi* (IgM 1 : 800, IgG negative), while the remaining workup was negative, including serology for other rickettsiae. Therefore, doxycycline 100 mg twice per day was added and ceftriaxone with metronidazole was stopped.

The patient improved within 72 hours of doxycycline treatment, with resolution of fever and abdominal pain and normalization of liver enzymes and ultrasound findings. He had a full recovery and was discharged on day 8 with a diagnosis of possible murine (endemic) typhus with instructions to complete a 10-day course of oral doxycycline (100 mg twice per day). A repeat IFA test during his follow-up visit 14 days after discharge demonstrated a marked increase in antibody titers for* R. typhi* (IgM, 1 : 51,200; IgG, 1 : 960), thus confirming the diagnosis of murine typhus.

## 3. Discussion

Infections caused by* R. typhi* usually have an uncomplicated self-limited course and are typically associated with low mortality rates [1–4]. However, a wide range of clinical manifestations and occasionally severe forms of infection are reported [1]. Most cases evade diagnosis because of nonspecific symptoms, including fever, chills, malaise, headache, and rash [1]. In addition, because of the self-limited course, it is likely underdiagnosed in endemic areas. Therefore, diagnosis is mostly based on clinical suspicion, local epidemiology, patient exposures, and characteristic laboratory abnormalities which include leukopenia, thrombocytopenia, mild to moderate elevation of hepatic enzymes, hypoalbuminemia, and electrolyte disturbances, mainly hypocalcemia and hyponatremia [1]. Confirmation of the diagnosis of murine typhus relies primarily on serological or molecular methods [2]. In cases of suspected cross-reactivity between the various typhus group agents (primarily,* R. typhi* and* R. prowazekii*), differences in titers against these microorganisms could be of help [6].

Apart from the aforementioned common clinical features, abdominal symptoms in murine typhus such as nausea, vomiting, and abdominal tenderness are not unusual [2, 4]. Furthermore, serious clinical presentations often mimicking surgical emergencies, such as cholecystitis and appendicitis, have been reported [7]. Nevertheless, as rickettsial disease is part of the differential diagnosis of acute febrile disease, a high index of suspicion is required to avoid further unnecessary interventions [8]. In our case, prompt and correct diagnosis led to successful treatment of the patient with antibiotics. As a result, cholecystectomy, which would be unnecessary, was avoided.

To the best of our knowledge, only one case of murine typhus and acalculous cholecystitis has been previously reported, concerning a patient who developed RUQ pain and macular rash following a course of prolonged fever [8]. Ultrasonography revealed acalculous cholecystitis. The patient was successfully treated with ampicillin-gentamicin-metronidazole and was discharged with a plan for elective cholecystectomy. However, because of his recent travel history, serology sent for* R. typhi* on admission documented the diagnosis of murine typhus and cholecystectomy was avoided [8]. Another study reported a patient with sepsis, abdominal pain, and liver enzyme abnormalities due to murine typhus, who underwent urgent cholecystectomy for presumptive diagnosis of acalculous cholecystitis [7]. However, histopathological examination of the resected gallbladder failed to confirm the diagnosis [7].

Acalculous cholecystitis usually occurs in patients who are critically ill and hospitalized. It typically complicates surgery, burn injury, major trauma, and shock of any cause but has been also correlated with congestive heart failure, diabetes mellitus, other chronic debilitating diseases, embolization of the cystic artery, total parenteral nutrition, mechanical ventilation, immunosuppression, and abdominal vasculitis [9]. These conditions lead to ischemia of the gallbladder wall resulting in a local inflammatory response [9].

The primary target of rickettsiae in humans is the endothelial cells, leading to vasculitis which may affect any organ and yield generalized or localized clinical and laboratory findings [1, 10]. Histopathological findings of the gallbladder in two patients with acute cholecystitis and* R. rickettsii* infection who underwent emergent cholecystectomy demonstrated typical lesions of rickettsial vascular injury, including perivascular and intramural inflammation and nonocclusive thrombosis [11]. Our patient was not critically ill or hypovolemic and we were unable to detect an overt cause of acalculous cholecystitis. Thus, in our case, based on the patient's medical history, the nature and clinical course of his disease, and response to doxycycline, it is reasonable to assume that acute acalculous cholecystitis was due to rickettsial vasculitis involving the gallbladder.

Reasons accounting for the variety of disease presentation and severity among different patients are not always clear, but male sex, older age, delayed appropriate treatment, hematologic disorders, and underlying hepatic or renal disease are recognized risk factors [1, 2, 7]. Our patient was heterozygous for *β*-thalassemia, a condition which has been previously described in patients with liver involvement in murine typhus [7]. Hyposplenism, as supported by the detection of Howell-Jolly bodies, may have also contributed to the severity of infection, but the cause of hyposplenism remains unknown.

## 4. Conclusion

In conclusion, we present a patient with* R. typhi* infection, manifesting with acalculous cholecystitis, who had an uneventful outcome following prompt initiation of doxycycline. The significance of this case lies in the correct diagnosis of this infrequent presentation of murine typhus, which was based on a high level of awareness due to local epidemiology, patient exposures, and clinical and laboratory findings, obviating an unnecessary surgical intervention.
